# Diclofenac Degradation—Enzymes, Genetic Background and Cellular Alterations Triggered in Diclofenac-Metabolizing Strain *Pseudomonas moorei* KB4

**DOI:** 10.3390/ijms21186786

**Published:** 2020-09-16

**Authors:** Joanna Żur, Artur Piński, Danuta Wojcieszyńska, Wojciech Smułek, Urszula Guzik

**Affiliations:** 1 Institute of Biology, Biotechnology and Environmental Protection, Faculty of Natural Sciences, University of Silesia in Katowice, Jagiellońska 28, 40-032 Katowice, Poland; apinski@us.edu.pl (A.P.); danuta.wojcieszynska@us.edu.pl (D.W.); 2 Institute of Chemical Technology and Engineering, Poznan University of Technology, Berdychowo 4, 60-695 Poznan, Poland; wojciech.smulek@put.poznan.pl

**Keywords:** biotransformation enzymes, cells injury, diclofenac, gene expression, membranes, metabolites, oxidative stress, *Pseudomonas*, toxicity

## Abstract

Diclofenac (DCF) constitutes one of the most significant ecopollutants detected in various environmental matrices. Biological clean-up technologies that rely on xenobiotics-degrading microorganisms are considered as a valuable alternative for chemical oxidation methods. Up to now, the knowledge about DCF multi-level influence on bacterial cells is fragmentary. In this study, we evaluate the degradation potential and impact of DCF on *Pseudomonas moorei* KB4 strain. In mono-substrate culture KB4 metabolized 0.5 mg L^−1^ of DCF, but supplementation with glucose (Glc) and sodium acetate (SA) increased degraded doses up to 1 mg L^−1^ within 12 days. For all established conditions, 4′-OH-DCF and DCF-lactam were identified. Gene expression analysis revealed the up-regulation of selected genes encoding biotransformation enzymes in the presence of DCF, in both mono-substrate and co-metabolic conditions. The multifactorial analysis of KB4 cell exposure to DCF showed a decrease in the zeta-potential with a simultaneous increase in the cell wall hydrophobicity. Magnified membrane permeability was coupled with the significant increase in the branched (19:0 *anteiso*) and cyclopropane (17:0 *cyclo*) fatty acid accompanied with reduced amounts of unsaturated ones. DCF injures the cells which is expressed by raised activities of acid and alkaline phosphatases as well as formation of lipids peroxidation products (LPX). The elevated activity of superoxide dismutase (SOD) and catalase (CAT) testified that DCF induced oxidative stress.

## 1. Introduction

Diclofenac (DCF), a phenylacetic acid derivative classified as a non-steroidal anti-inflammatory drug (NSAID), due to wide prevalence is considered as one of the most important contaminants of emerging concern. The presence of DCF in drinking [1,2,3], ground [4,5] or surface water [6,7], as well as sewage [8] and activated sludge [9,10] ranged from 0.02 ng L^−1^ to 20 μg L^−1^ [2]. The estimated annual worldwide consumption of DCF is around 2400 tons, which is partly conditioned by its over-the-counter access [3]. In recent years, the usefulness of traditional physico-chemical sewage treatment methods applied to the removal of recalcitrant pollutants is being increasingly questioned [2]. Despite that removal efficiency of DCF by advanced oxidation processes reaches up to 80%, the limitations of these methods frequently preclude their use [10,11,12]. DCF as a hydrophobic chlorinated derivative with electron-withdrawing and donating groups has a log D < 3.2 at pH 8.0, which hinders the biological removal [13,14]. Bioremediation is an eco-safe and economically justified alternative for harsh chemical treatments being based on the individual microbial strain able to detoxify the particular compound or microbial consortia with more complex metabolic properties. Bacteria from the genus *Pseudomonas* are widely known as able to reside in hostile environments since they constitute the indigenous microflora of various contaminated areas i.e., soil, sludge, water bodies and wastewater. The ecological success of pseudomonads can be attributed to great metabolic versatility, effective detoxifying and adapting mechanisms based on the high oxidoreductase activities, adjusting the membranes fluidity or extensive transport, and secretion systems [15].

To date, a few reports about complete DCF degradation have been published (Table S1). Moreira et al. [10] described *Labrys portucalensis* F11 strain able to degrade DCF under the co-metabolic conditions with sodium acetate. Ivshina et al. [2] isolated *Rhodococcus ruber* IEGM 346 strain able to degrade 50 μg L^−1^ of DCF during 6 days. The bacterial DCF degradation has also been demonstrated for *Enterobacter hormachei* D15 [16], *Raoultella* sp. DD4 [17], *Brevibacterium* sp. D4 [18] and *Klebsiella* sp. KSC [19]. More recently, Palyzová et al. [20] with the use of chemical mutagenesis obtained a *Raoultella* sp. KDF8 strain that is capable of converting DCF to primary metabolic products. Nguyen et al. [21] reported about the potential of bacteria from genera: *Nitratireductor*, *Asticcacaulis* and *Pseudoxanthomonas* to metabolize DCF under co-metabolic conditions. Finally, Grandclément et al. [14] described *Bacillus subtilis* and *Brevibacillus laterosporus* that metabolized 1 mg L^−1^ of DCF in a nutrient broth medium during 17 h. Navrozidou et al. [22] described the main microbiota involved in DCF-degradation in the activated sludge fed with DCF as a sole carbon and energy source. The major bacterial taxa identified in the immobilized cell biofilter were *Granulicella pectinivorans* and *Rhodanobacter terrae* as well as the members of the species *Castellaniella denitrificans*, *Parvibaculum lavamentivorans*, *Bordetella petrii*, *Bryocella elongata* and *Rhodopseudomonas palustris*. We have previously described two novel strains isolated from DCF-treated activated sludge from *Serratia* and *Rahnella* genera [23]. So far, degradation of DCF has not been confirmed for any strain of *Pseudomonas* genus. Despite the relatively large body of the literature about DCF degradation, mainly by activated sludge microflora [18,23,24,25,26], the genetic background of its decomposition remains scarce. Palyzová et al. [20] reported about the presence of few genes related to DCF degradation encoding catechol 1,2-dioxygenase, protocatechuate 3,4-dioxygenase and quercetin 2,3-dioxygenase in *Raoultella* sp. KDF8 strain genome. The genetic analysis coupled with shot-gun proteomics revealed the up-regulation of 24 intracellular proteins engaged in the metabolic pathways of degradation of benzoate, catechol and genes associated with oxidoreductases in response to DCF treatment.

The native microflora play an important role in the biological detoxification of contaminants acting as a primary response triggering the modifying reactions. Considering the literature data, there is a limited understanding of DCF exposure effects on prokaryotic organisms, thus broadening of knowledge about protective mechanisms and the influence of xenobiotics on bacteria is still necessary [2]. The impact of DCF on the physiological status of the cell is mostly multidirectional and concerns different cellular and sub-cellular levels i.e., oxidative stress, lipids peroxidation, alterations in receptor activity, destruction of the membrane-associated proteins or membranes permeability modification [27,28]. The two most frequently detected DCF hydroxylated derivatives: 4′-OH-DCF and 5-OH-DCF, can be further oxidized to reactive benzoquinone imines that interplay with the protein nucleophilic groups, which results in adducts formation [2,27,29]. Physico-chemical properties of DCF molecule, mainly the *n*-octanol/water partition coefficient (log K_ow_, 4.51), are responsible for potential bioaccumulation in the living organisms, mainly aquatic microbiota. The ecotoxicological effects of DCF even at concentration levels as low as 1 μg L^−1^ have been reported for different groups of organisms, e.g., *Daphnia magna* where exposure resulted in the multiplied formation of reactive oxygen species (ROS), decreased filtration and ingestion rates, leading to behavioral and biochemical alterations [3,14]. The high toxicity of DCF at environmentally relevant concentrations on duckweed *Lemna minor* was reported by Kummerová et al. [30]. Fu et al. [31] investigated the interplay between bioaccumulation and biotransformation processes of DCF in the model invertebrates *Gammarus pulex* and *Hyalella azteca*. The recently described DCF by-product, DCF-methyl ester was characterized by a significantly higher bioconcentration factor, namely 25- and 110-fold for *G. pulex* and *H. azteca*, respectively. The LC_50_ of DCF for *H. azteca* was amounted at 216 mg L^−1^, while that of methyl ester was reduced to 0.53 mg L^−1^, indicating a 430-fold increase in acute toxicity compared to DCF [31]. The most known example of toxicity of DCF residues is the decline of the population of oriental black-white vultures in India and Pakistan due to renal failure [32].

Considering the gaps of knowledge in the aspect of DCF bacterial degradation, this study aimed to (i) analyzing the catabolic potential of *Pseudomonas moorei* KB4 to DCF degrade under different nutritional conditions and secondary metabolites formation, (ii) measuring DCF degradation-related relative gene expression and (iii) estimating the multidirectional influence of DCF on the bacterial cells and oxidative stress level triggered by DCF. Despite the literature data about DCF influence on the bacterial cells, holistic studies about its interplay with DCF-degrading strains including gene expression analysis, degradative enzymes and a battery of physiological assays are scarce.

## 2. Results

### 2.1. Degradation and Metabolites Formation of DCF under Monosubstrate and Co-Metabolic Conditions

**Figure 1:**
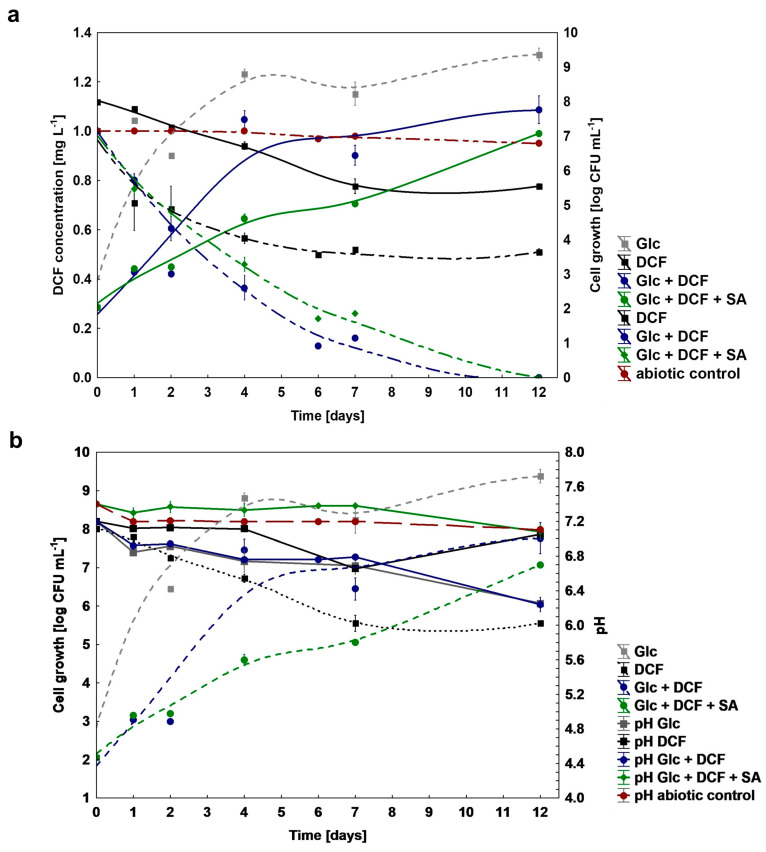
(**a**) Changes in cell growth (log CFU mL^−1^) and degradation of DCF under mono-substrate and co-metabolic conditions; (**b**) changes in the pH during DCF degradation. DCF—diclofenac, Glc—glucose, SA—sodium acetate.

To test a catabolic potential of the KB4 strain to DCF degradation, different nutritional conditions were established. Based on the obtained results, the KB4 strain was characterized by different catabolic capabilities dependent on the conditions which were applied (Figure 1a). In the mono-substrate experiment, the degraded dose of DCF amounted to 0.51 ± 0.01 mg L^−1^ (51%) for 12 days. Under co-metabolic conditions with Glc supplementation, the degradation was most effective and reached 1 mg L^−1^ (100%) within 11 days. The similar efficiency of DCF degradation amounted to 1 mg L^−1^ (100%) was achieved by the addition of two additional carbon sources, namely Glc and SA. However, degradation time for this system was extended by one day, compared to co-metabolic culture with Glc. The lack of significant changes in the concentration of DCF within 12 days in the abiotic control without bacteria confirmed no chemical oxidation of DCF. The average degradation rate was 0.045 ± 0.00, 0.142 ± 0.00 and 0.125 ± 0.00 mg L^−1^ day^−1^ for mono-substrate, supplemented with Glc and supplemented with Glc and SA cultures, respectively. For all established cultures target analysis using UHPLC/MS/MS and/or GC-TOFMS of secondary metabolites formation was performed (Figure S1). Based on the literature data about the most frequently occurring microbial DCF by-products, we confirmed the formation of 4′-hydroxylated DCF (4′-OH-DCF) and generated during intramolecular ring closure DCF-lactam in both mono-substrate and co-metabolic cultures (Figure S1). Additionally, for co-metabolic culture with Glc, the degradation product with *m/z* at 41, 55, 75, 149 and 207 corresponding to phthalate acid was found. The measurements of degradation were coupled with cell growth and pH estimation (Figure 1b). Despite the observed degradation of DCF in mono-substrate culture, the increase in the number of cells for this system was not observed, which indicated that the KB4 is not able to proliferate in the presence of DCF as a sole carbon and energy source., The most significant cell proliferation has been observed for co-metabolic conditions with Glc and for Glc and SA. In the control experiment with Glc cell growth was substantially higher, which suggested high toxicity of DCF. The pH measurements indicated that Glc assimilation lowered pH value. For the control, the initial pH dropped from the initial 7.20 ± 0.00 to the final 6.25 ± 0.06. Over the experiment, pH of the culture in the mono-substrate system ranged from the initial 7.20 ± 0.00 to 7.05 ± 0.01 at the end of the experiment and starting from 7.20 ± 0.00 to 6.24 ± 0.08 at the end for the co-metabolic conditions with Glc, and from 7.40 ± 0.00 to 7.08 ± 0.08 for Glc and SA.

### 2.2. Analysis of the Specific Activity of Enzymes Involved in DCF Degradation and Its Genetic Background

**Figure 2:**
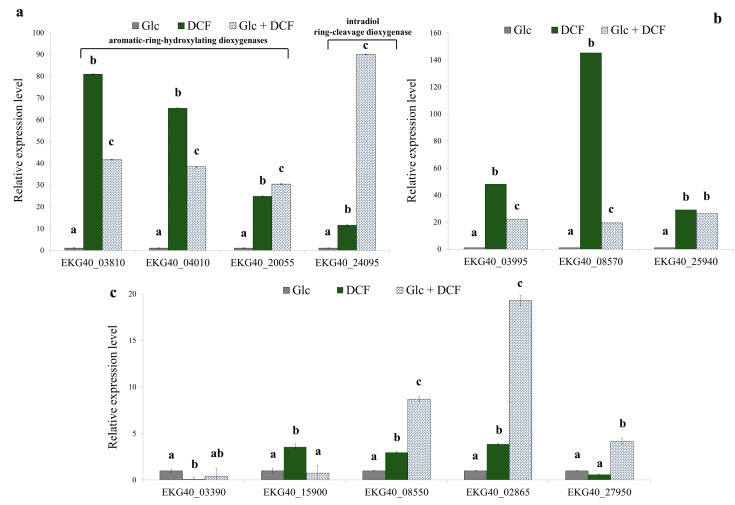
The relative expression level of the selected genes found in the KB4 genome. (**a**) Aromatic-ring hydroxylating dioxygenases (EKG40_03810, EKG40_04010, EKG40_20055), intradiol ring cleavage-dioxygenase (EKG40_24095); (**b**) catechol 1,2-dioxygenases (EKG40_03995, EKG40_08570 and EKG40_25940); (**c**) deaminases (EKG40_03390 and EKG40_15900), benzoate 1,2-dioxygenase (EKG40_08550), homogentisate 1,2-dioxygenase (EKG40_02865) and cytochrome P-450 (EKG40_27950), which were measured using the RT-qPCR technique with *gyrA* (EKG40_13720) as the reference gene. The data are presented as the mean ± SD of six replicates; differences in the relative expression level were determined with a one-way ANOVA followed by the Tukey’s test; different letters denote significant differences (*p*-value < 0.05) between the treatment.

In order to reveal the enzymology of microbial degradation of DCF, the specific activity of selected enzymes from different groups was measured (Table 1). For enzymes with identified corresponding genes in the KB4 genome, the relative expression levels were determined (Figure 2). Thus, the specific activity of 16 enzymes and the expression of 13 genes for control, mono-substrate and co-metabolic cultures was measured. The expression level of two considered aromatic-ring-hydroxylating dioxygenases (EKG40_03810 and EKG40_04010) increased in DCF treatment in mono-substrate culture. The highest up-regulation in the expression level was observed in the dioxygenase (EKG40_03810), in which expression was increased 80-times compared to the control treatment. In the co-metabolic culture, the up-regulation was estimated at almost 42-fold. In the mono-substrate culture, EKG40_04010 gene expression was 65-fold higher than the control treatment with Glc. For co-metabolic culture with Glc + DCF, the expression of the EKG40_04010 increased almost 38-fold compared to the control treatment. The third analyzed gene (EKG40_20055) was characterized by the highest activity observed in the co-metabolic conditions.

The measurements of the specific activity of dixydroxylating dioxygenase showed a similar trend with the highest activity observed for mono-substrate culture. The expression level of two analyzed catechol 1,2-dioxygenases, EKG40_03995 and EKG40_08570, significantly increased after DCF exposure, 48 and 145-times, respectively. In the presence of Glc and DCF, the up-regulation was less pronounced and amounted to 19-fold for EKG40_03995 and 22-fold for EKG40_08570. The expression of the third catechol 1,2-dioxygenase (EKG40_25940) showed up-regulation after DCF treatment but leveled-off for both conditions. The expression level of the intradiol ring-cleavage dioxygenase (EKG40_24095) was almost 11.5-fold higher in the presence of DCF. The highest up-regulation (almost 90-fold) was measured for the co-metabolic conditions with DCF and Glc. The expression of deaminase (EKG40_15900) was significantly up-regulated in the presence of DCF. A quite similar level was observed in the control with Glc, and co-metabolic culture with Glc and DCF. The highest activity of deaminase in the mono-substrate culture with DCF was observed. The expression of second gene encoding deaminase (EKG40_15900) was down-regulated in both treatments with the highest drop observed in co-metabolic conditions. The expression of benzoate 1,2-dioxygenase (EKG40_08550) was almost 3-times higher for mono-substrate culture than the value measured for control with Glc. The significant up-regulation (above 8.5-times) of this gene was observed in co-metabolic culture. The expression of homogentisate 1,2-dioxygenase (EKG40_02865) in the culture with Glc and DCF was almost 20-fold and 5-fold higher than expression measured for the control with Glc and mono-substrate culture with DCF, respectively. Similar results were obtained from the enzymatic measurements, were the highest activity of homogentisate 1,2-dioxygenase was observed in co-metabolic culture. The expression of the gene that encodes cytochrome P-450 (EKG40_27950) increased 4-fold in co-metabolic conditions. For the mono-substrate culture, the expression was almost twice lower than the control. For the other enzymes, in the case of the aromatic monooxygenase measured with phenol as a substrate the highest specific activity was observed for co-metabolic culture. Similarly, the highest activity of protocatechuate 3,4-dioxygenase and salicylate 1,2-dioxygenase was observed in co-metabolic culture. The opposite trend, with the highest activity in mono-substrate culture, was observed for hydroquinone 1,2-dioxygenase, quercetin 2,3-dioxygenase and laccase. No activity was detected for gentisate 1,2-dioxygenase and protocatechuate 4,5-dioxygenase. Two enzymes were active only in the control with Glc, namely, catechol 2,3-dioxygenase and hydroxyquinol 1,2-dioxygenase.

### 2.3. Zeta Potential and Cell Wall Hydrophobicity Analysis

**Figure 3:**
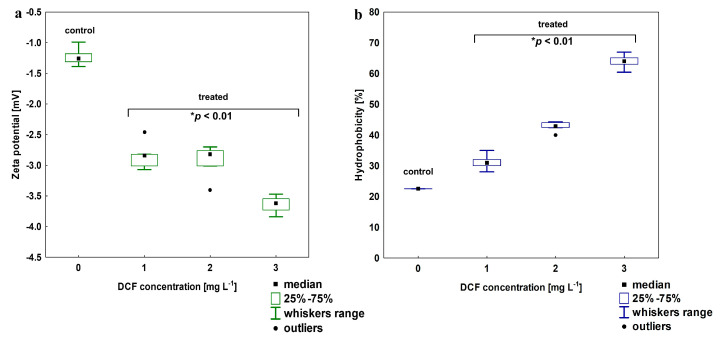
(**a**) Changes in zeta potential for DCF-treated cells at a concentration of 1, 2 and 3 mg L^−1^; (**b**) changes in the cells wall hydrophobicity in DCF-treated cells at the same range of concentration. For all treatments, the observed changes between treated and control cells were statistically significant (one-way ANOVA followed by the least significant differences (LSD) test with a *p*-value < 0.01).

The electro-surface properties of the KB4 cells after exposure to increasing DCF concentration were estimated by zeta-potential measurements (Figure 3a). For the KB4 cells non-treated with DCF zeta-potential was estimated at −1.22 ± 0.15 mV. After exposure to 1 mg L^−1^ DCF, the decrease to −2.84 ± 0.23 mV was observed. The elevation of DCF concentration resulted in the further decline of zeta- potential value, which was estimated for 2 mg L^−1^ and 3 mg L^−1^ as −2.93 ± 0.23 and −3.42 ± 0.48 mV, respectively. All of the zeta-potential values observed after DCF treatment were statistically different than the value measured for the control sample (*p* < 0.01). For cell wall hydrophobicity analysis, a strong trend was observed—cell wall hydrophobicity increased with DCF concentration (Figure 3b). The KB4 cells cultivated on MSM are characterized by relatively low hydrophobicity (22.54 ± 2.01%) as we described previously [33]. After DCF treatment, the hydrophilicity of the KB4 strain cell wall significantly declined (*p* < 0.01) and the cell wall became more hydrophobic reaching up to 63.94 ± 2.43% for the highest tested DCF concentration of 3 mg L^−1^.

### 2.4. The Influence of DCF on Fatty Acids Composition and Membrane Permeability

**Figure 4:**
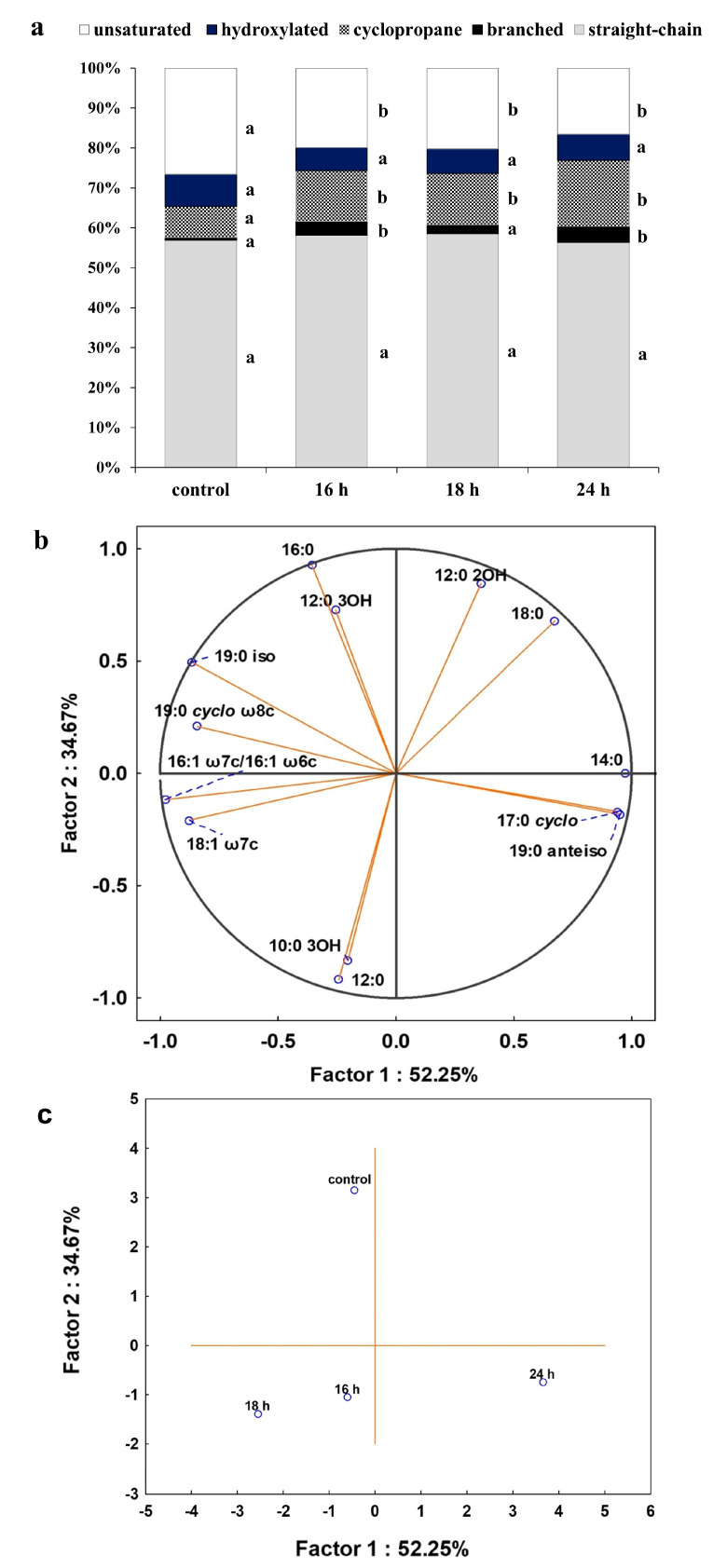
(**a**) Percentage of total fatty acids for control and cells after DCF exposure at the concentration of 1 mg L^−1^ after different incubation times. The data are presented as the mean values of three biological replicates; (**b**) principal component analysis (PCA) of fatty acid ratio in the control and cultures with the addition of DCF; (**c**) the projection of fatty acids profiles isolated from the control and DCF-treated cells after different incubation times on the plane defined by Factor 1 and Factor 2. a,b,c—statistically significant differences using a one-way ANOVA with a *p*-value < 0.05 followed by least significant differences (LSD) test.

**Figure 5:**
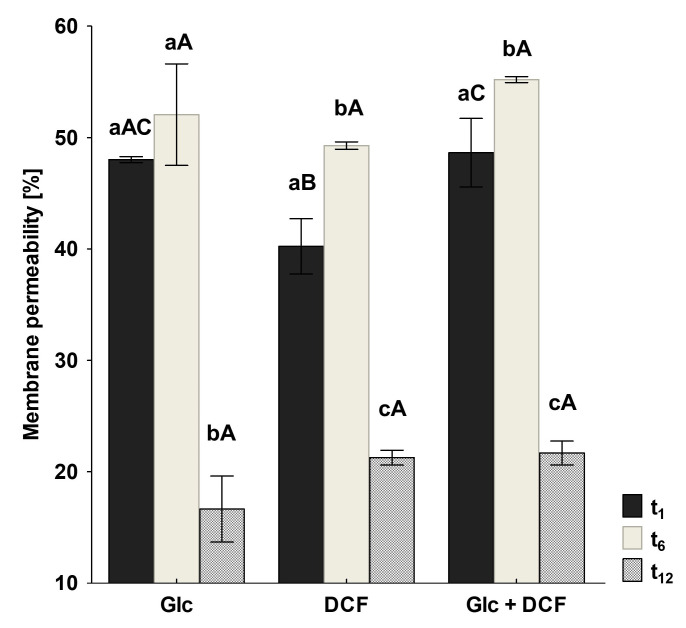
Changes in membrane permeability measured by CV uptake. CV—crystal violet, DCF—diclofenac, Glc—glucose. The data are presented as the mean values of three biological replicates ± standard deviation. a,b,c—statistically significant differences using a one-way ANOVA with a *p*-value < 0.05 followed by least significant differences (LSD) test for different times; A,B,C—statistically significant differences using a one-way ANOVA with a *p*-value < 0.05 followed by least significant differences (LSD) test for different treatments.

As membrane lipids are one of the most adaptable molecules, alterations in their composition and amount can be a valuable marker of the toxic effects of DCF on bacterial cells. In this study, identified fatty acids were divided into two groups: unsaturated and saturated. The latter was further divided into hydroxyl, straight-chain and branched ones. Analysis of the obtained profiles showed relevant changes in the content of unsaturated, 16:1ω6c/16:1ω7c and 18:1ω6c/18:1ω7c acid, and cyclopropane fatty acids between control samples with Glc and after DCF treatment for all selected times of incubation (Figure 4). The statistically significant changes were also observed in the amount of branched fatty acids, 19:0 *anteiso* and 19:0 *iso*, between control and DCF-treated samples after 16 h and 24 h of incubation. Analysis of the content of straight-chain and hydroxylated fatty acids showed no significant changes between cells exposed to Glc and DCF. Among cyclopropane fatty acids, the highest content of 17:0 *cyclo* was observed after the shortest exposure time of 16 h. The presence of 19:0 *cyclo* ω8c was characterized only for DCF-treated samples. The level of hydroxylated acid content, mainly 10:0 3OH and 12:0 3OH, was rather constant and not exceeds 3.5%. The principal component analysis (PCA) (Figure 4) suggested that fatty acid methyl esters (FAMEs) extracted from DCF-treated samples after 24 h were distinct in comparison with the other fatty acids along with the factor 1 (52.25%) and were mainly characterized by diverse content of 12:0 2OH, 18:0, 14:0, 17:0 *cyclo* and 19:0 *anteiso* acids. The analysis of the fatty acids composition was coupled with the membrane’s permeability measurements. A similar trend was observed for all DCF treatments and control culture with Glc (Figure 5). In the control samples, the lowest CV uptake which indicated low permeability of 48.03 ± 0.25% was noticed in sixth day of the experiment (t_6_). At the start of the experiment (t_1_), the permeability was slightly higher and amounted to 52.06 ± 4.55%. On the last day of the experiment (t_12_), the permeability was significantly altered and estimated at 16.64 ± 2.96%. Over the experiment, the lowest permeability was observed for mono-substrate culture with DCF. At the beginning of treatment (t_1_), the permeability reached 40.23 ± 2.48% with a slight increase to 49.29 ± 0.33% observed at t_6_. The final value in the 12 day dropped from 27.60 ± 5.03 to 21.25 ± 0.66%. For co-metabolic culture with Glc and DCF, the similar observations concerned the changes in the permeability were made. The lowest CV uptake was reported at t_6_ (55.20 ± 0.26%), with quite a similar level of 48.65 ± 3.06% at t_1_ and the lowest permeability measured at the end of the experiment (21.67 ± 1.06%).

### 2.5. Oxidative Stress and Cells Injury Induced by DCF

The level of oxidative stress induced by DCF treatment was measured by catalase (CAT) and superoxide dismutase (SOD) activity. The CAT activity in DCF treatment for mono-substrate and co-metabolic conditions increased comparing to the control with Glc (Table 2). Statistically lower activity compared to co-metabolic culture was measured for mono-substrate conditions. The lowest SOD activity was measured for the control sample with Glc. Slightly higher activity was observed for Glc + DCF, whereas for mono-substrate culture, the SOD activity raised almost 6.5-fold. The cell’s injury was estimated by measuring the activity of phosphatases with different pH optimum. The up-regulation of the activity of alkaline phosphate compared to the control with Glc was observed for all established cultures with the highest value observed for the mono-substrate conditions. For acid phosphatase, under mono-substrate conditions, the activity was lower than the basal activity in the presence of Glc. The slight increase was observed for co-metabolic culture with the highest activity of 0.34 ± 0.02 Bessey’s units. LPX induced by the presence of DCF was observed for both mono-substrate and co-metabolic cultures, with the highest malondialdehyde (MDA) concentration observed in DCF and Glc culture.

## 3. Discussion

Due to the increasing concerns about environmental pollution, in recent years the broadening of knowledge about the multi-level influence of xenobiotics on bacterial strains used in clean-up technologies is observed and realized in a growing number of isolated strains able to DCF metabolize [14]. Up to now, several DCF-degrading strains belonging to different genera including Gram-negative *Klebsiella* sp., *Pseudoxanthomonas* sp., *Raoultella* sp. or *Serratia* sp., as well as Gram-positive *Bacillus* sp., *Brevibacillus* sp., *Enterococcus* sp. or *Rhodococcus* sp., have been described. In this study, we aimed to estimate a catabolic potential of *P. moorei* KB4 strain, partly conditioned by its high tolerance (EC_50_ above 5.0 g L^−1^) towards DCF under different metabolic conditions. The achieved degraded doses ranged between 0.5–1.0 mg L^−1^ for both, mono-substrate and co-metabolic cultures supplemented with Glc and Glc + SA, far exceeds the environmentally relevant concentrations of DCF and its metabolites. However, the time required for degradation indicated high chemical stability of the DCF molecule [2]. For most DCF-degrading isolates, co-metabolic conditions with various additional carbon sources i.e., Glc, SA or glycerol, have been applied. It is widely known that supplementation with an external source of carbon and energy may ensure bacterial growth and, subsequently, the better degradation capabilities [34]. For the KB4 strain, the addition of Glc and SA generally allow to intensified DCF degradation from 51% to 100% of the administered dose. Similar observations were made by Ivshina et al. [2], who showed the intensification of DCF degradation in the presence of Glc and Moreira et al. [10], who achieved higher degraded doses of DCF by periodic supplementation of the culture with SA. However, the presence of easily accessible carbon source, which serves as an electron-donating compound, may have also the opposite effect as demonstrated by Larcher et al. [35] and Aissaoui et al. [16]. A similar degraded dose was shown by Grandclément et al. [14] for *B. subtilis* and *B. laterosporus*, which metabolized 1 mg L^−1^ of DCF in a nutrient broth medium during 17 h. In our previous work, we have isolated from DCF-treated activated sludge *S. proteamaculans* AS4 and *R. bruchi* AS7 strains, that were able to degrade nearly 0.5 mg L^−1^ of DCF within 14 days after 1.0 g L^−1^ of Glc addition [23]. Moreira et al. [10] described complete degradation of DCF via co-metabolism at the concentration of 1.7 µM during 6 days and 34 µM during 25 days by *L. portucalensis* F11 strain. Aissaoui et al. [16] achieved 52.8% elimination of DCF at the concentration of 10 mg L^−1^. After Glc addition, degraded dose increased up to approximately 82%. The significantly higher degradation rate was shown by Palyzová et al. [36] for *Raoultella* sp. KDF8 strain after chemical mutagenesis which metabolized nearly 1.0 g L^−1^ of DCF. A lack of significant (2–5%) physico-chemical DCF decomposition observed within this study, which confirmed the biological nature of DCF degradation, was also showed by Ivshina et al. [2]. During the experiment, the cell growth measurements were performed. For mono-substrate culture, the decrease in the bacterial biomass was observed, which indicates that DCF at the applied concentration was insufficient as the sole carbon and energy source. For the control experiment with Glc, the observed number of cells was significantly higher than those measured for co-metabolic cultures which may indicate DCF toxicity. Interestingly, for culture with two external carbon sources, Glc and SA, the growth was significantly lower in the period of 2 to 9 days in comparison with culture with DCF and Glc. Moreover, no significant reduction in degradation rate was observed in the presence of SA. In addition, after 10 days of culture, the growth in both co-metabolic systems was comparable. This may indicate differences in DCF metabolism by the tested strain in the presence of SA. To complete the overall picture of DCF degradation, the pH measurements of cultures were also performed. A similar trend was observed for all established cultures, but with a varied dynamic. It is widely known that Glc assimilation lowers the pH, mainly due to acetoin and organic acids formation [36,37], thus, the strongest decrease in the pH was observed for control with Glc. In the mono-substrate culture, the pH slightly decreases to almost neutral (7.05 ± 0.01). Noteworthy, DCF was applied as a sodium salt which additionally may alter the pH of the culture. Pobudkowska and Domańska [38] indicated that the pKa of DCF sodium ranges between 4 and 5.7. This indicates its dissociation under culture conditions, which leads to slight alkalization of the environment. At the same time, during DCF degradation, the formation of phthalic acid was observed. The influence of these two phenomena may explain the pH value observed at the end of the culture. Among co-metabolic cultures, the greater decline was observed for Glc + DCF system which may indicate that for the system with DCF + Glc + SA, SA alleviated the pH value. The alkaline or almost neutral pH may also be a result of by-products formation, mainly amines e.g., 4-aminophenol or 4-amino-3,5-dichlorophenol formed e.g., via amidation or amide hydrolysis, thus alkalizing the culture [2,26]. On the other hand, Palyzová et al. [36] demonstrated that after 96 h of degradation, the pH of the culture with basal salts broth medium supplemented with DCF dissolved in ethanol and incubated at 28 °C ranged from 3.4 to 4.6. Low pH of the cultures may also be associated with the formation of carbonic acid. The literature data regarding the optimal pH for DCF degradation are inconsistent. According to Vieno and Sillanpää [9], the acidic pH enhanced DCF removal, nevertheless, Palyzová et al. [36] for *Raoultella* sp. KDF8 strain demonstrated that in the initial pH below 7, DCF degradation is impeded, and most of its dose remained undissolved, thus, authors pointed out that the optimal pH for DCF removal varied from 7 to 9.

The reactions occurring during DCF degradation are mostly multidirectional [10,25,26]. The major advance of our results to previous reports where several identified metabolites and proposition of the putative DCF pathway were given, rely on the measurements of the specific activity of degradative enzymes and integration of these data with relative expression level of genes encoding those enzymes. Most recently, Ivshina et al. [2] proposed three putative DCF degradation pathways with 16 identified metabolites on the example of *R. ruber* IEGM 346 strain. The hydroxylation of DCF molecules to 4′-OH-DCF, which is considered as a bottleneck metabolite during DCF biodegradation, up to now was confirmed also for *Actinoplanes* sp. [39], *Raoultella* sp. KDF8 [36] and *L. portucalensis* F11 strains [10]. The target analysis performed within this study for the KB4 strain confirmed the formation of 4′-OH-DCF in all established cultures, which indicated that the initial steps of DCF biodegradation proceed via hydroxylation and seem to be common for both mono-substrate and co-metabolic conditions. Noteworthy, 4′-OH-DCF formation is also observed during human metabolism of DCF by the activity of cytochrome P-450, which was also shown for bacteria [14]. The high expression of gene encoding cytochrome P-450 during DCF degradation by the KB4 strain confirmed its involvement in DCF metabolism. The second identified metabolite whose formation was proved for all cultures —DCF-lactam—was also found during DCF degradation by *E. hormaechei* D15 and the microbial consortia in the activated sludge [26]. Interestingly, in all studies regarding DCF degradation, several additional metabolites with defined *m/z* spectra have been found, however some were unidentified and their chemical structures were unresolved [9,40]. Further degradation of hydroxylated derivatives may proceed via different pathways, which is indirectly proven by the activity of dioxygenases cleaving the aromatic ring differently. As a result of hydroxylation, four key metabolites may be formed: catechol, protocatechuic acid, hydroxyquinol, and gentisic acid [41]. For the latter intermediate, we did not observe the activity of dedicated dioxygenase. The low activity of 1,2-hydroxyquinol dioxygenase was observed for cells from the mono-substrate culture. Interestingly, the activity was lower than this observed for the control cells. Intermediates analysis revealed the presence of phthalic acid which may be further degraded into 4,5-dihydroxyphthalate, which was suggested to be formed by dioxygenation of phthalate to 4,5-dihydro-4,5-dihydroxyphthalate followed by decarboxylation to key metabolite—protocatechuate. This key metabolite may be cleaved by protocatechuate dioxygenases or converted to catechol [42,43]. Our results showed inhibition of protocatechuate 3,4-dioxygenase by DCF and lack of protocatechuate 4,5-dioxygenase activity. The lack of the activity of catechol 2,3-dioxygenase in DCF-treated cells and simultaneous high activity of catechol 1,2-dioxygenase indicated that the by-products are *ortho* cleaved to *cis*,*cis*-muconic acid. The formation of by-products without the aromatic structure allows to their further incorporation into the Kreb’s cycle. Moreover, the activity of homogentisate 1,2-dioxygenase in the presence of DCF was observed. It is possible that one of DCF intermediates is homogentisate. This intermediate may be formed by hydrolysis of the amino bond and hydroxylation of DCF ring A. The activity of homogentisate 1,2-dioxygenase may indicate the formation of homogentisic (2,5-dihydroxyphenylacetic) acid also found by Ivshina et al. [2] during DCF degradation by *R. ruber* IEGM 346 strain. For this strain, homogentisic acid was further transformed into 2-(*p*-benzoquinone-2)acetic acid. The presence of gene encoding quercetin 2,3-dioxygenase in the genome of *Raoultella* sp. KDF8 strain was confirmed by Palyzová et al. [20]. Herein, we confirmed the low activity of this enzyme in DCF-treated cells in the mono-substrate culture. This enzyme is known to catalyze the oxidative decomposition of quercetin to 2-protocatechuoylphloroglucinol carboxylic acid and carbon monoxide [44]. 2-protocatechuoylphloroglucinol carboxylic acid may be further transformed to 2,4,6-trihydroxycarboxylic acid, e.g., benzoic acid. The contribution of quercetin 2,3-dioxygenase as well as salicylate 1,2-dioxygenase in DCF degradation by strain KB4 is unclear. The proteomic analysis performed by Palyzová et al. [20] revealed the engagement of proteins assigned to benzoate degradation cluster into DCF degradation. Our study confirmed the activity of both enzyme and gene encoding benzoate 1,2-dioxygenase during DCF degradation by the KB4 strain. Benzoate 1,2-dioxygenase catalyzes cleavage of benzoate to 1,6-dihydroxycyclohexa-2,4-diene-1-carboxylate, which may be further transformed to catechol. Very low activity of hydroquinone 1,2-dioxygenase, peroxidase and laccase indicate that those enzymes are not significantly involved in the bacterial DCF degradation. Measurement of ammonia concentration indicates deaminase activity, which is also confirmed by the results of deaminase gene expression analysis. Worth to note is the significantly higher ammonia concentration detected for mono-substrate culture, compared to co-metabolic and control with Glc. Moreover, appearance of a monocyclic intermediate of DCF also confirms the role of the amino acid hydrolyzing enzyme in DCF degradation.

The surface charge of bacteria at neutral pH is negative mainly due to the complex structure of the cell wall that contains anionic surface groups [45]. The exposure of the KB4 cells on the increasing DCF concentrations resulted in the significant (*p* < 0.01) changes in the electro-surface properties measured by zeta-potential in the control and DCF-treated cells. A gradual decrease in zeta-potential was observed at the examined DCF concentration ranged from 0 to 3 mg L^−1^. A similar trend was observed by Ivshina et al. [2] who exposed cells of *R. ruber* IEGM 346 on high (50 mg L^−1^) and environmentally relevant concentrations (50 µg L^−1^) of DCF. Despite that, at the first stage of the experiment, the shift of zeta-potential value was observed, which was attributed to the cationic nature of DCF sodium salt and its interplay with mycolic acid –COOH groups present in the rhodococcal cell wall [2]. In our study, the shift towards the positive value was not observed. Probably, the negatively charged phthalate ion formed during degradation outweighs the balance between the negatively charged components of the bacterial cell membrane and the cationic nature of DCF sodium. This leads to a significant increase in negative charge at the interface. Despite that, during bacteria cultivation, the pH of the culture is subject to change and the number of the involutional and lysed cells increased which is also reflected in the general cell’s potential. For the same range of DCF concentration as for zeta-potential measurements, the cell wall hydrophobicity estimation was performed. The cells of the KB4 strain unexposed to ecopollutants cultivated in the minimal medium were characterized by low hydrophobicity measured by the microbial adhesion to hydrocarbons (MATH) test [32]. Similarly to the study performed on rhodococci [2], the cell wall of the KB4 after DCF exposure became more hydrophobic. The interaction between DCF molecules with the cells’ compounds boosted cell aggregation mainly through the elevated hydrophobicity. The decrease of zeta-potential and shift of the cell wall to being more hydrophobic was also observed by Yalçin et al. [46] for *P. putida* exposed on polycationic cetyltrimethylammonium bromide surfactant and Mohanty and Mukherji [47] for Triton X-100 treated *Burkholderia* sp. strain. Both compounds possess a cationic character similar to DCF sodium molecule. Moreover, all these compounds are characterized by a hydrophobic nature. On the other hand, no simple correlation between alteration in zeta-potential and hydrophobicity was observed by Smułek et al. [48] for *Rahnella* sp. EK12 strain after long-term contact with saponins and rhamnolipids.

The presence of xenobiotics in the environment may result in severe changes in the liquidity and integrity of the cell membrane [49,50]. From the environmental point of view and the potential usage of the KB4 strain in bioremediation processes, the lack of significant alterations at as high as 1 mg L^−1^ of DCF concentration in the lipid composition is beneficial. However, for all tested time points, a significant increase in the cyclopropane and branched fatty acids (without 18 h) was observed. The properties of the membrane depend on the ratio of the straight-chain and branched fatty acids as well as the saturated and unsaturated one [51]. The negative influence of aromatic pharmaceuticals on the example of ibuprofen on bacterial membrane properties was shown by Marchlewicz et al. [50]. In the presence of ibuprofen at the concentration of 2.0 g L^−1^, high content of branched fatty acids, mainly 18:0 *anteiso*, and a low amount of unsaturated fatty acids were identified. Herein, after DCF exposure, we observed the same trend, but a crucial branched fatty acid with the highest content after 24 h of incubation was identified as 19:0 *anteiso*. The increased content of branched fatty acids with a simultaneous high content of long-chain fatty acids modulates membranes’ fluidity through the increase of the temperature phase transition, which results in the decrease of membrane permeability [50,52]. A similar tendency regarding the elevated content of the branched fatty acids was reported by Unell et al. [53] for *Arthrobacter chlorophenolicus* A6 strain grown on phenol and its chloro- and nitro- derivatives. Górny et al. [54] investigated the impact of naproxen (other polycyclic NSAIDs) on the fatty acids composition on the example of Gram-positive *Bacillus thuringiensis* B1(2015b) strain. The presence of naproxen at a concentration as high as 4.8–5.2 g L^−1^ causes severe alterations in the membrane composition, i.e., the presence of the 16:0 *iso* 3OH acid only in the samples cultivated with naproxen, the slight decrease in the content of 18:0 *anteiso* acid at the highest tested concentration and remarkable decrease of the 18:1 ω9c acid. Palyzová et al. [55] described the alterations of lipid profile after DCF exposure in the time ranging from 24 to 72 h at a concentration of 1.0 g L^−1^ observed for *Raoultella* sp. KDF8 strain. The most significant changes concerned shifting from the mono-unsaturated to cyclopropane fatty acids, particularly 16:1 against 17:0 *cyclo*, which resulted in the geometric rearrangement of membranes. Authors hypothesized that due to those changes, strain is able to deal with destabilization of the inner and outer membrane. Moreover, decreased amounts of phosphatidylethanolamine with prolonged incubation time, and increased amounts of phospholipids having three or four acyls i.e., acyl-phosphatidylglycerol and cardiolipin with the prolonged time of cultivation, were observed [15,51,55].

Intracellular redox status of the bacterial cell can be altered by different environmental stimuli leading to the oxidative stress and subsequently, cell death [56]. The level of oxidative stress can be evaluated by various systems based on the measurable biochemical, morphological or physiological alterations caused by the toxic agents. In this study, the cells injury and oxidative stress triggered by DCF treatment were estimated by analysis of SOD and CAT activities, by measuring the concentration of LPX as well as by the activity of alkaline and acid phosphatases, which are responsible for the dephosphorylating reactions [28]. High values of SOD activity obtained in mono-substrate culture with DCF correlated with MDA concentrations clearly indicate oxidative stress caused by the presence of DCF. In the presence of Glc, with much more intense cell metabolism, probably DCF and its toxic metabolism products are rendered harmless much faster. On the other hand, low CAT activities values are surprising, both in mono-substrate and co-metabolic culture. Similar effect was observed by Zhang et al. [57] during a study on the response of antioxidant enzymes in *E. coli* K12 and *B. subtilis* B19 to atrazine stress. Results obtained for alkaline phosphate activity showed that the comparable level was observed for both, mono-substrate and co-metabolic cultures. Since the activity of phosphatases is strictly dependent on the pH of the cultures, its increased activity observed in mono-substrate conditions is consistent with the slightly basic pH observed during mono-substrate degradation. The high activity observed in the co-metabolic culture with the slightly acidic pH was less expected. For the control with Glc, the significantly lower activity indicated that the observed activity for the rest systems was induced by DCF presence. Acid phosphate with the pH optimum ranging from 3.5–7.0 [58] showed low activity for all tested conditions with the highest drop observed for mono-substrate culture characterized by the most alkaline pH. The elevated activity of alkaline phosphate after DCF treatment was reported by Chouchan and Sharma [59] in serum and skeletal muscle in mice. The sub-chronic exposure in the last two stages of treatment was presumably attributed to the detrimental effect of DCF on tissues, mainly by the affecting of transmembrane permeability of DCF, especially considering the involvement of the enzyme into adsorption and transport across the membranes. The alkaline phosphate mode of action was also connected to replacing by DCF the zinc atom in the active site of the enzyme. The accumulation of LPX measured by MDA concentration after DCF exposure coupled with the intensified oxidative stress met the criteria assumed by Bagnyukova et al. [60], who postulated that the formation of LPX is strictly involved in the intensification of enzymes which activity is characterized for oxidative stress, i.e., CAT. The correlation between the formation of LPX and CAT activity was shown by Saucedo-Vence et al. [61], who proved the simultaneous increase in the CAT activity and the concentration of LPX in common carp exposed to DCF at the concentration of 7 mg L^−1^. On the contrary, Gómez-Oliván [62], who studied the oxidative stress in the presence of DCF in *Daphnia magna* at the concentration of 1 g L^−1^, showed exceeding 90% increase in lipid peroxidation compared to control values, but CAT activity remained unchanged. Quite low concentrations of MDA formed for all examined treatments could indicate the low involvement of cytochrome P-450 in the biotransformation of DCF by the KB4 strain. However, despite the low concentration of MDA in the co-metabolic culture, the cytochrome P-450 gene expression was up-regulated. On the other hand, down-regulation observed in the mono-substrate culture was not reflected in the MDA concentration. For eukaryotes, the formation of the highly unbalanced oxygenated by-product the oxy-cytochrome P-450 complex has been observed, which may induce lipid peroxidation by releasing the superoxide anion [27,63]. Moreover, Feito et al. [64] observed the hormesis after 90 min of DCF exposure manifested by the decrease in LPX in *Danio rerio* embryos. At the higher concentration than 0.03 µg L^−1^ of DCF, LPX increased reaching the so-called zone of compensation. Hypothetically, under these conditions, the hormetic stimulus fails to compensate for the damage reduction and subsequently, the divergences between biomarker levels in the examined samples and controls are indeterminate.

## 4. Materials and Methods

### 4.1. Bacterial Strain

The object of this study was *P. moorei* KB4 isolated from the activated sludge previously described as paracetamol-degrading strain [33,65].

### 4.2. Degradation Experiments

Degradation experiments were performed at 30 °C in mineral salts medium (MSM) with the following composition: g L^−1^: 3.78 Na_2_HPO_4 ×_ 12 H_2_O; 0.5 KH_2_PO_4_; 5.0 NH_4_Cl; 0.2 MgSO_4_ × 7 H_2_O and 0.01 yeast extract under (1) mono-substrate conditions with 1 mg L^−1^ of DCF and initial OD_600_ set at 0.750, (2) co-metabolic conditions with glucose at a concentration of 0.1% (*v*/*v*), 1 mg L^−1^ of DCF and initial OD_600_ set at 0.05, (3) co-metabolic conditions with glucose (0.1%, *v*/*v*) and sodium acetate at the concentration of 5.6 mM, 1 mg L^−1^ of DCF and initial OD_600_ set at 0.05. The degradation rate was determined with the High-Performance Liquid Chromatography (HPLC) technique using Merck Hitachi reversed-phase chromatograph (Mannheim, Germany), equipped with a column Ascentis Express^®^ C18, pre-column Opti-Solv^®^ EXP and UV/Vis DAD detector. The mobile phase consisted of acetonitrile, methanol and 1% acetic acid (50:30:20, *v*/*v*, flow-rate 1 mL min^−1^). The detection wavelength was set at 276 nm. DCF in the supernatant was identified by comparing the HPLC retention time and UV spectra with that of the external standard [23].

### 4.3. Secondary Metabolites Identification

Identification of the secondary metabolites formed during DCF degradation in each treatment was performed using liquid chromatography in reverse phase system coupled with a mass detector (UHPLC/MS/MS) Shimadzu LC/MS-8040, using a Luna Omega C18 1.6 μm column (100 × 2.1 mm, Phenomenex, (Torrance, CA, USA) and mobile phase consisted of: A—5 mM aqueous solution of ammonium acetate, pH 5.8, B—acetonitrile. The mobile phase flow was 0.4 mL min^−1^ and the oven temperature was 30 °C. The elution program was as follows: 0–2 min—10% isocratic B, 2–9 min from 10 to 70% B, 9–10 min—isocratic 70% B, 10–10.5 min—from 70 to 5% B, 10.5–14 min—isocratic 5% B. Mass spectrometer parameters were: nebulizing gas flow 3 L min^−1^, drying gas flow 8 L min^−1^, temperature 250 °C desolvation line, 400 °C heating block temperature. The analyses were performed in multiple reaction monitoring (MRM) mode [23]. For gas chromatography the samples were prepared according to Marchlewicz et al. (2017). Prior to analysis, to each sample 0.1 mL of derivatization reagent MTBSTFA with 1% t-BDMCS was added. After incubation at 60 °C for 30 min, the samples were dissolved in 1 mL of hexane. The qualitative analysis of metabolites was performed on PEGASUS 4D GCxGC-TOFMS gas chromatograph (LECO Corp., St. Joseph, MI, USA) connected to a BPX5 (5% phenyl equivalent, 28 m × 0.25 mm; 0.25 μm) capillary column (SGE Int., Melbourne, Australia). The ion source and transfer line temperature were set at 250 °C. Helium was used as carrier gas, with a flow of 1.0 mL min^−1^. After splitless injection of 1 μL of the sample, the oven temperature was set and maintained for 2 min at 40 °C, then it was applied a heating ramp to 300 °C at a rate of 12 °C/min and the temperature of the oven was maintained for 15 min. The ionization source was operated in the positive ion mode (electron energy: 70 V) and the acquisition rate was set at 10 spectra/s [66].

### 4.4. Analysis of the Specific Activity of Selected Enzymes Involved in DCF Degradation

For enzymes activity assay culture of the KB4 strain exposed to 1.0 mg L^−1^ of DCF was harvested by centrifugation (5000× *g*, 4 °C, 20 min), the pellet was washed with 50 mM of phosphate buffer (pH 7.2) and re-suspended in the same buffer. Cell extract was prepared by sonication (6 times for 15 s, with 30 s breaks with frequency of 20 kHz) of the whole-cell suspension and centrifuged (10,000 × *g*, 4 °C, 30 min). The obtained supernatant was used as a crude extract for further enzyme assays. Aromatic monooxygenase, catechol 1,2-dioxygenase, catechol 2,3-dioxygenase, protocatechuate 3,4-dioxygenase, protocatechuate 4,5-dioxygenase, gentisate 1,2-dioxygenase, hydroxyquinol and hydroquinone 1,2-dioxygenase, laccases and peroxidases were assayed as was previously described by Marchlewicz et al. [66]. The activity of the dioxygenase catalyzed dihydroxylation was determined according to Wojcieszyńska et al. [67]. The activity of salicylate 1,2-dioxygenase was measured according to Górny et al. [54]. Quercetin 2,3-dioxygenase was measured according to Schaab et al. [44], benzoate 1,2-dioxygenase was measured according to Wolfe et al. [68]. Homogentisate 1,2-dioxygenase was determined according to Sariaslani et al. [69]. Deaminase was determined according to Żur et al. [65].

### 4.5. Analysis of Expression of Selected Genes Encoding Enzymes Involved in Microbial Degradation of DCF

For gene expression analysis total RNA was isolated from bacteria cultivated under mono- and co-metabolic conditions using a GeneMATRIX Universal RNA Purification Kit (EURx, Gdansk, Poland). The RNA concentration was quantified by measuring the absorbance at 260 nm using an ND-1000 NanoDrop spectrophotometer (Thermo Scientific, Waltham, MA, USA). The residual genomic DNA was digested using RNase-free DNase (Invitrogen Life Technologies, Carlsbad, CA, USA) and the purity of the RNA samples was assessed at the absorbance ratios of OD_260/280_ and OD_260/230_, while its structural integrity was determined using agarose gel electrophoresis. Single-stranded cDNA was synthesized from 1 μg of the total RNA using a RevertAid First Strand cDNA Synthesis Kit (Thermo Scientific, Waltham, MA, USA) according to the manufacturer’s instructions. The generated cDNA was used for the RT-qPCR reactions using a LightCycler^®^ 480 SYBR Green I Master (Roche, Basel, Switzerland)in a LightCycler^®^ 480 Real-Time PCR System (Roche, Basel, Switzerland). All of the PCR reactions were performed in 96-wells Multiwell Plates under the following conditions: 10 min at 95 °C and 45 cycles of 15 s at 94 °C, 30 s at 60 °C and 30 s at 72 °C. Three biological replicates and two technical replicates were used. The gene encoding gyrase (*gyrA*) was used as internal control [33]. The relative expression level was calculated according to Livak and Schmittgen [70]. Primers that were specific for the selected genes were designed using Geneious Prime (version 2019.0.3) and are listed in Table S2.

### 4.6. Zeta Potential Measuring

The zeta potential was calculated from the Smoluchowski equation following measurements of electrophoresis mobility using the ZetaPlus instrument (Brookhaven Instruments Co., Holtsville, NY, USA). The changes in zeta potential were measured after the exposure of the cells at 1, 2 and 3 mg L^−1^ of DCF. Bacterial cells from the exponential growth phase were centrifuged (8000× *g*, 5 min) and washed twice with MSM to remove residual of DCF and then suspended in the same medium to a final concentration of 10^8^ CFU mL^−1^ [71,72].

### 4.7. Cell Wall Hydrophobicity Measurements

Cell wall hydrophobicity measurements were performed as described by Kos et al. [73] and Nachtigall et al. [74]. The adherence was calculated from the following equation, (1 − A_t_)/A_0_ × 100, where A_t_ means the absorbance at time 15 min and A_0_ the absorbance at time 0.

### 4.8. Fatty Acid Extraction and Analysis

The fatty acid composition of KB4 strain was determined after 16 h, 18 h and 24 h of cultivation in lysogeny broth (LB) medium (control sample), and LB medium containing DCF at the concentration of 1 mg L^−1^. Briefly, bacteria were harvested by centrifugation (5000× *g*, 4 °C, 20 min) and the cell pellets were washed twice with 0.9% NaCl to remove the residue of the culture medium. The fatty acid isolation and identification were conducted by the MIDI–MIS method [51]. Three replicates of data obtained from each treatment were analyzed statistically by one-way ANOVA. The statistical significance (*p* < 0.05) of the differences was assessed by a post hoc comparison of the means using the least significant difference (LSD) test. The FAMEs profiles were also subjected to principal component analysis (PCA). All analyses were performed using the Statistica 13.0 PL software. To examine the changes in FAMEs profiles of bacteria dependent on DCF expose and prevent the alterations caused by fatty acids that were only detected occasionally, the analysis of the FAMEs included only fatty acids with a content of at least 1% in the FAMEs profile.

### 4.9. Membrane Permeability Analysis

Changes in membrane permeability were estimated by crystal violet (CV) assay according to Pacholak et al. [72]. Briefly, bacteria were harvested in the exponential phase, centrifuged (5000× *g*, 20 min, 4 °C) and washed with MSM. Biomass was re-suspended in MSM to fit the optical density approximately 1.0. Next, 0.95 mL of obtained cell suspension was mixed with 50 µL of CV solution (0.1 mg mL^−1^). Samples were incubated at 30 °C for 10 min and centrifuged (13,400× *g*, 15 min). The optical density of the supernatant (A_sample_) was measured at a wavelength of 590 nm. The OD of the CV solution mixed with MSM at the same ratio (A_violet_) was considered as 100%. The permeability expressed as the percentage of CV uptake was calculated according to the equation:

(1) $$\mathrm{uptake}\;\mathrm{of}\;\mathrm{crystal}\;\mathrm{violet}\;\%\;=\;\frac{A_{\mathrm{violet}}-{\;A}_{\mathrm{sample}}}{A_{\mathrm{violet}}}\times 100\%$$

### 4.10. Cells Injury and Oxidative Stress Analysis

The activity of alkaline and acid phosphatase was determined by the formation of *p*-nitrophenol at 415 nm for both crude extracts (intracellular) and in the supernatant (extracellular). Catalase activity was estimated by measuring the decrease in absorbance at 240 nm with 54 mM H_2_O_2_. The activity of superoxide dismutase was measured using SOD Assay Kit No 19160-1KT-F (Merck, Darmstadt, Germany) according to the manufacturer’s instructions. One unit (U) of the enzyme activity was defined as the amount of enzyme required to generate 1 µmol of product per minute. Protein concentration in the crude extract was determined by the Bradford method [66]. The activity of alkaline and acid phosphatase was calculated in Bessey units, denoting the amount of mmol of *p*-nitrophenol released by the enzyme in 1000 mL of the enzyme fraction during 1 h of incubation. To determine the lipids peroxidation to 1 mL of crude extract, 1 mL of trichloroacetic acid (15%, *w/v* in 0.25 M HCl) and 1 mL of thiobarbituric acid (0.37%, *w*/*v*, in 0.25 M HCl) were added. Afterward, the mixtures were incubated at 100 °C for 10 min. Samples were left to cool down, centrifuged (5000× *g*, 4 °C, 20 min) and measured spectrophotometrically at 535 nm. Two control samples were prepared, one by adding 1 mL of water instead of crude extract, and the second by adding 1 mL of water instead of thiobarbituric acid [75]. The concentration of lipids peroxidation products was calculated based on the malonate aldehyde molecular extinction coefficient ε = 156,000/cm^−1^ M^−1^.

### 4.11. Statistical Analysis

All of the data were expressed as the mean value and standard deviation of three biological replicates. Data were analyzed using Microsoft Office Excel 2010 and Statistica^®^ 13.0 PL (TIBCO Software Inc., Palo Alto, CA, USA). To test the normality of the data distribution the Shapiro–Wilk (*p* < 0.05) test was applied. The statistical significance (*p* < 0.05 or *p* < 0.01) of any differences were analyzed using one-way ANOVA and evaluated by a post-hoc test of the means using the lowest significant differences (LSD) test.

## 5. Conclusions

*P. moorei* KB4 was able to degrade 0.5 mg L^−1^ of DCF in mono-substrate and 1 mg L^−1^ in co-metabolic culture with Glc and SA within 12 days. For all established cultures, two key DCF metabolites have been identified, i.e., 4′-OH-DCF and DCF-lactam. The specific activity of enzymes putatively involved in DCF bacterial degradation confirmed the multidirectional character of DCF metabolism, including the formation of hydroxylated derivatives and aliphatic by-products without aromatic structure. The up-regulation of selected genes encoding degradative enzymes, i.e., monooxygenase, dihydroxylating dioxygenases, cleavage dioxygenases or cytochrome P-450, in DCF-treated cells testified about their involvement in the metabolic pathway of DCF. The exposure of the KB4 cells on DCF caused a decrease in zeta-potential with a simultaneous increase in the cell wall hydrophobicity. These changes were also coupled with a rise in the membrane’s permeability and alterations in the fatty acids’ composition. The increase in the branched and cyclopropane fatty acids was connected with a reduced amount of unsaturated ones. The elevated activity of SOD and CAT indicated that DCF triggered oxidative stress.

## Figures and Tables

**Table 1:** The specific activity of selected enzymes (U mg^−1^ of protein) measured for control with Glc, mono-substrate conditions with DCF and co-metabolic conditions with Glc and DCF.

Enzyme	Glc	DCF	Glc + DCF
Aromatic monooxygenase	3.065 ± 0.001 ^a^	11.660 ± 0.006 ^b^	110.115 ± 0.07 ^c^
Dihydroxylating dioxygenase - naphthalene	0.000 ± 0.000 ^a^	12.012 ± 0.001 ^b^	6.940 ± 0.043 ^c^
Salicylate 1,2-dioxygenase	0.000 ± 0.000 ^a^	0.207 ± 0.071 ^b^	0.302 ± 0.021 ^c^
Gentisate 1,2-dioxygenase	0.000 ± 0.000	0.000 ± 0.000	0.000 ± 0.000
Homogentisate 1,2-dioxygenase	0.000 ± 0.000 ^a^	1.034 ± 0.002 ^b^	3.006 ± 0.004 ^c^
Catechol 1,2-dioxygenase	1.000 ± 0.010 ^a^	2.062 ± 0.051 ^b^	1.731 ± 0.035 ^c^
Catechol 2,3-dioxygenase	1.850 ± 0.094 ^a^	0.000 ± 0.000 ^b^	0.000 ± 0.000 ^b^
Protocatechuate 4,5-dioxygenase	0.000 ± 0.000	0.000 ± 0.000	0.000 ± 0.000
Protocatechuate 3,4-dioxygenase	55.123 ± 0.010 ^a^	12.249 ± 0.010 ^b^	66.695 ± 0.189 ^c^
Hydroquinone 1,2 dioxygenase	0.000 ± 0.000 ^a^	0.004 ± 0.000 ^b^	0.000 ± 0.000 ^a^
Hydroxyquinol 1,2-dioxygenase	0.932 ± 0.046 ^a^	0.108 ± 0.025 ^b^	0.000 ± 0.000 ^c^
Benzoate 1,2-dioxygenase	0.000 ± 0.000 ^a^	1.395 ± 0.000 ^b^	3.033 ± 0.000 ^c^
Quercetin 2,3-dioxygenase	0.000 ± 0.000 ^a^	0.023 ± 0.001 ^a^	0.000 ± 0.000 ^a^
Peroxidase	0.017 ± 0.002 ^a^	0.004 ± 0.001 ^b^	0.004 ± 0.002 ^b^
Laccase	0.204 ± 0.152 ^a^	0.221 ± 0.058 ^a^	0.028 ± 0.011 ^b^
Deaminase	6.172 ± 0.023 ^a^	16.138 ± 0.045 ^b^	4.756 ± 0.018 ^c^

a,b,c—statistically significant differences using a one-way ANOVA with a *p*-value < 0.05 followed by least significant differences (LSD) test.

**Table 2:** The specific activity of CAT (U mg^−1^ of protein), SOD (inhibition rate, %), LPX (MDA, nmol mg^−1^ of protein), alkaline and acid phosphatases (Bessey’s units).

Enzyme/Parameter	Glc	DCF	Glc + DCF
CAT	0.30 ± 0.11 ^a^	1.04 ± 0.06 ^b^	2.34 ± 0.57 ^c^
SOD	10.51 ± 0.01 ^a^	80.31 ± 0.00 ^b^	12.59 ± 0.03 ^c^
alkaline phosphatase	0.02 ± 0.01 ^a^	2.10 ± 0.03 ^b^	2.07 ± 0.03 ^b^
acid phosphatase	0.17 ± 0.01 ^a^	0.04 ± 0.00 ^b^	0.34 ± 0.02 ^c^
LPX	0.00 ± 0.00 ^a^	8.67 × 10^−6 b^	9.87 × 10^−8 c^

CAT—catalase, SOD—superoxide dismutase, LPX—lipid peroxidation products, MDA—malondialdehyde, Glc—glucose, DCF—diclofenac, SA—sodium acetate. a,b,c—statistically significant differences using one-way ANOVA with a *p*-value < 0.05 followed by the least significant differences (LSD) test.

## Supplementary Materials

Supplementary Materials can be found at https://www.mdpi.com/1422-0067/21/18/6786/s1. Table S1. The overview of DCF bacterial metabolism. Table S2: The oligonucleotide primers that were used for the RT-qPCR reactions with the relevant description of the genes. Figure S1: Chromatograms of DCF and putative metabolites identified via UHPLC MS/MS and spectra identified via GC/MS analyses.

## Abbreviations

DCF	Diclofenac
FAME	Fatty acid methyl ester
Glc	Glucose
SA	Sodium acetate
OD	Optical density
SOD	Superoxide dismutase
CV	Crystal violet
ANOVA	Analysis of variance
HPLC	High-Performance Liquid Chromatography
UHPLC/MS/MS	Ultra-High Performance Liquid Chromatography coupled with Mass Spectrometry
LB	Lysogeny broth
MSM	Mineral salts medium
mV	Millivolts
4′-OH-DCF	4′-hydroxylated diclofenac
5-OH-DCF	5-hydroxylated diclofenac
DCF-lactam	Lactam diclofenac
DAD	Diode array detector
LSD	Least significant differences test
CFU	Colony forming unit
