# Supplementary material for: Diclofenac Degradation—Enzymes, Genetic Background and Cellular Alterations Triggered in Diclofenac-Metabolizing Strain Pseudomonas moorei KB4
Source: Int J Mol Sci. 2020 Sep 16;21(18):6786. doi: 10.3390/ijms21186786 (PMC7555183; doi:10.3390/ijms21186786)
Supplement: Supplementary file 1 [file ijms-21-06786-s001.pdf]

a

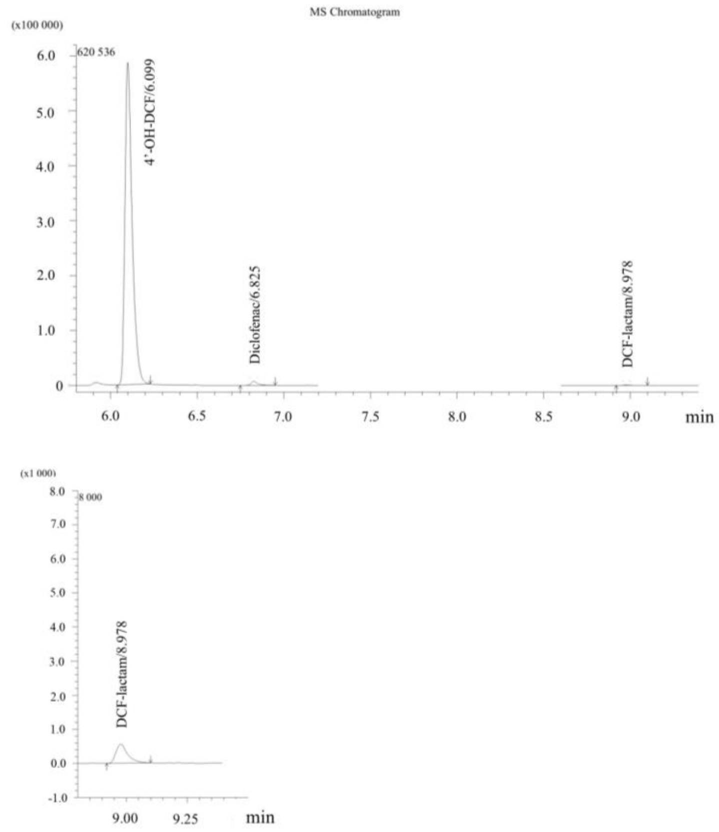

MS Chromatogram
(x100 000)
620 536
4'-OH-DCF/6.099
Diclofenac/6.825
DCF-lactam/8.978
6.0
6.5
7.0
7.5
8.0
8.5
9.0
min
(x1 000)
8 000
DCF-lactam/8.978
9.00
9.25
min

b

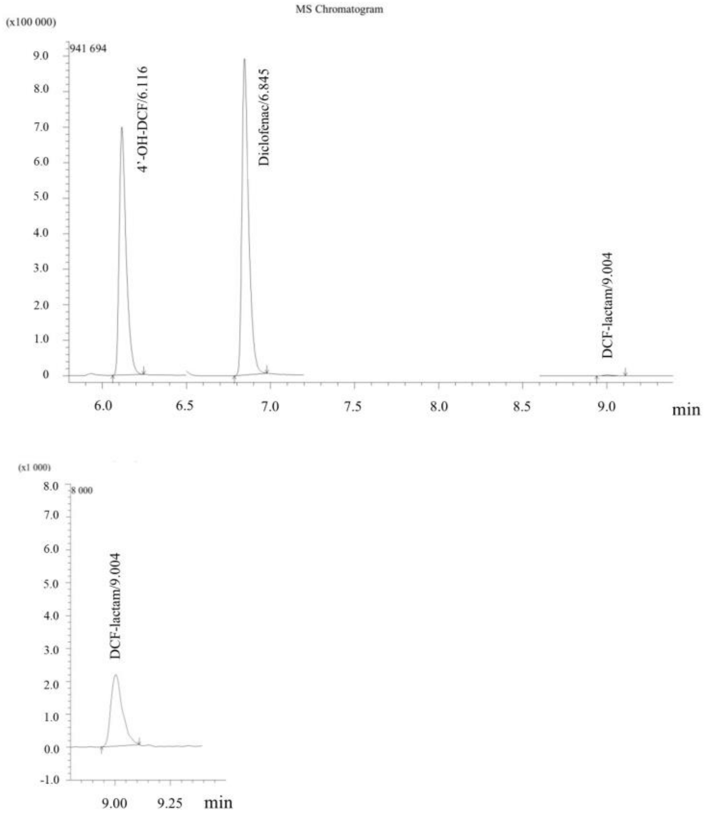
MS Chromatogram
(x100 000)
941 694
4'-OH-DCF/6.116
Diclofenac/6.845
DCF-lactam/9.004
min
(x1 000)
8 000
DCF-lactam/9.004
min

c

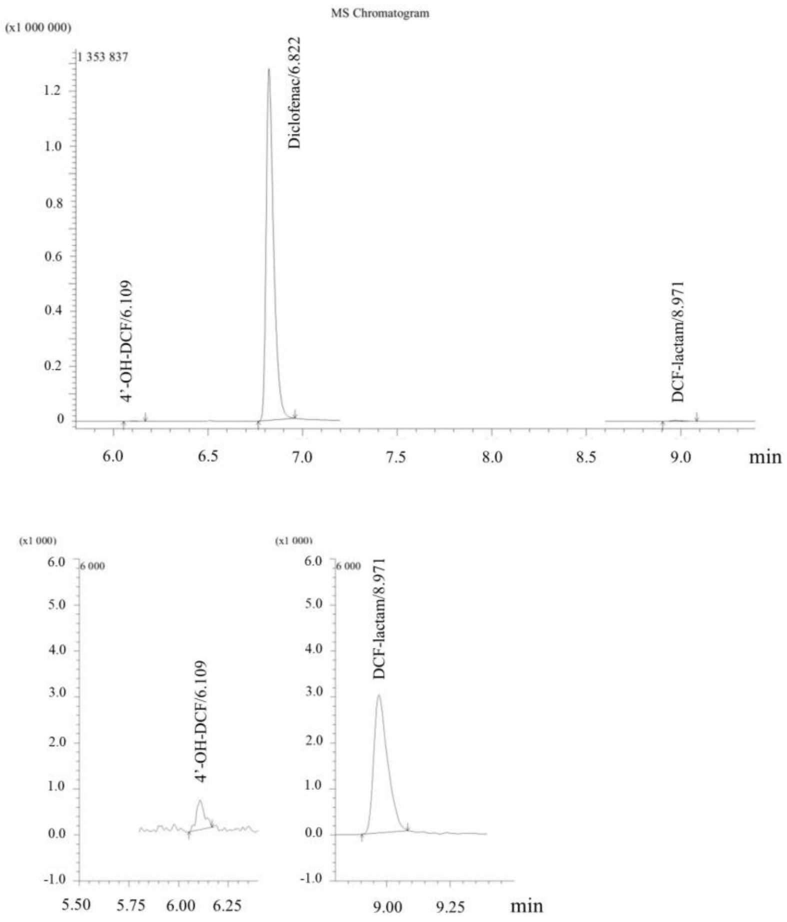

d

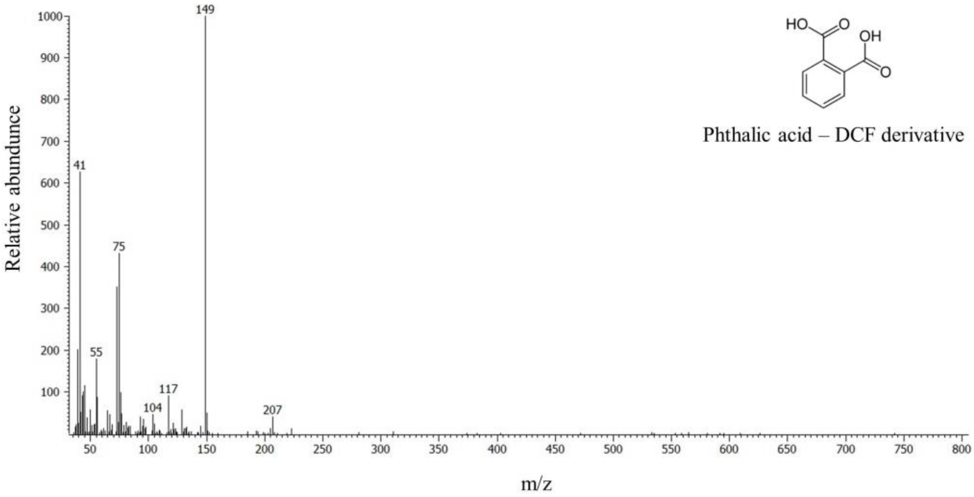

Figure S1. Chromatograms of DCF and putative metabolites identified *via* (**a-c**) UHPLC MS/MS and spectra identified *via* GC/MS analyses (**d**).

Table S1. The overview of DCF bacterial metabolism.

Strains	Metabolites	Putative enzymes/processes	Genes	References
Bacillus subtilis *Brevibacillus laterosporus*	4′-OH-DCF	nd	nd	[14]
Rhodococcus ruber IEGM 346	2-[2-(2′,6′ di-chloro-anilino)phenyl]acetic acid; 2-[2-(2′,6′-dichloro-4′-hydroxyanilino)phenyl]acetic acid; 2-[2-(2′,6′-dichloroanilino)-5-hydroxyphenyl]acetic acid; 2-(1-(5-oxo-cyclohexa-1,3-dienyl-2-(2′,6′-dichloro-phenylimino)acetic acid; 4-amino-3,5-dichlorophenol; phenylacetic acid, 5-amino-4,6-dichlorobenzene-1,2-diol; 3-hydroxyphenylacetic acid; 2,5-dihydroxyphenylacetic acid (homogentisic acid); 2-(*p*-benzoquinone-2)acetic acid; 4,6-dioxooct-2-*trans*-enedioic acid (fumarylacetoacetic acid); 3-oxobutanoic acid (acetoacetic acid); *trans*-butenedioic acid (fumaric acid); 4,6,7-trioxooct-2-enedioic acid; 2-[1-(5-oxocyclohexa-1,3-dienyl-2-(3′,4′-dihydroxy-2′,6′-dichlorophenyl)imino]acetic acid	oxidation, hydroxylation, ring-cleavage	nd	[2]
Raoultella sp. KDF8	18 compounds retained the structure of the core of the DCF molecule and 14 compounds had structures indicating	1,2-dioxygenase, protocatechuate, 3,4-dioxygenase,	nd	[20,36]

	subsequent DCF secondary amine bond cleavage. The most important metabolites: 4’-OH-DCF, 5-OH-DCF, 6-OH-DCF,(*E*)-4-((2,6-dichloro-4-hydroxyphenyl)imino) -3-(hydroxymethyl)cyclohexa-2,5-dien-1-one, (*E*)-(6-((2,6-dichloro-3,4-dihydroxyphenyl)imino)-3-oxocyclohexa-1,4-dien-1-yl)methanolate, 4-amino-3,5-dichlorobenzene-1,2-diol, 3,5-dichlorobenzene-1,2,4-triol, 2-(2,3-dihydroxyphenyl)acetic acid, 3-(carboxycarbonyl)pent-4-enoic acid, 3-(carboxycarbonyl)-2-hydroxypent-4-enoic acid, 3-(carboxycarbonyl)-2-oxopent-4-enoic acid, primary metabolites - malonic, oxoglutaric, 3-hydroxyglutaric, and hydroxy-oxo-valeric acids	quercetin 2,3-dioxygenase, phenylacetate-CoA ligase, 4-hydroxyphenylacetate catabolism protein, 3,4-dihydroxyphenylacetate 2,3-dioxygenase, 4-hydroxyphenylacetate 3-monooxygenase reductase subunit, muconolactone isomerase, muconate and chloromuconate cycloisomerase, 3-oxoadipate CoA-transferase, 2,3-dihydroxybenzoate-2,3-dehydrogenase, beta-ketoadipyl CoA thiolase, succinyl-CoA:3-ketoacid-coenzyme A transferase subunit B, succinyl-CoA synthetase subunit beta		
Actinoplanes sp. ATTC 53771	4’-OH-DCF, 5-OH-DCF	cytochrome P-450	*cyp107E4*	[39]
Enterobacter hormachei D15	1-(2,6-dichlorophenyl)-1,3-dihydro-2H-indol-2-one	nd	nd	[16]
Klebsiella sp. KSC	OH-DCF, 2-OH-DCF, 3-OH-DCF, 4-OH-DCF, and several transformation products without proposed structure	cytochrome P-450, hydroxylation, dihydroxylation, dehydrogenation, decarboxylation, cyclization, ring-cleavage	nd	[19]

Labrys portucalensis F11	Twelve metabolites with proposed structure	oxygenation, hydroxylation, dihydroxylation, methylation, decarboxylation	nd	[10]
Pseudomonas moorei KB4	4’-OH-DCF, lactam-DCF, phthalic acid	aromatic monooxygenase, dihydroxylating dioxygenase – naphthalene, salicylate 1,2-dioxygenase, gentisate 1,2-dioxygenase, homogentisate 1,2-dioxygenase, catechol 1,2-dioxygenase, catechol 2,3-dioxygenase, protocatechuate 4,5-dioxygenase, protocatechuate 3,4-dioxygenase, hydroquinone 1,2 dioxygenase, hydroxyquinol 1,2-dioxygenase, benzoate 1,2-dioxygenase, quercetin 2,3-dioxygenase, peroxidase, laccase, deaminase	EKG40_03810 (*arhd*) EKG40_04010 (*arhd*) EKG40_20055 (*arhd*) EKG40_24095 (*chqB*) EKG40_03995 (*catA*) EKG40_08570 (*catA*) EKG40_25940 (*catA*) EKG40_03390 (*codA*) EKG40_15900 (*tadA*) EKG40_08555 (*cbdB*) EKG40_02865 (*hgd*) EKG40_27950 (*cyp*)	This study

nd – not determined, OH - hydroxyl group, DCF – diclofenac, CoA- coenzyme A

Table S2. The oligonucleotide primers that were used for the RT-qPCR reactions with the relevant description of the genes.

Locus tag	Gene description	Forward (5'-3')	Reverse (5'-3')	Product lenght (bp)
EKG40_03810	aromatic-ring-hydroxylating dioxygenase	AATCAAACCGCACCTCAG	GCATGAGCCAATCGTAGTAG	91
EKG40_04010	aromatic-ring-hydroxylating dioxygenase	CTGCTGAGTCGCGTAATG	GATCTGCTTGACGACGATAC	101
EKG40_20055	aromatic-ring-hydroxylating dioxygenase	TGACTTGCGCATCGAAC	AGACGGAAACGCGAAAC	90
EKG40_24095	intradiol ring-cleavage dioxygenase	ACAACACCTCAGCAACAC	TTCGCTGATATTGCGTCTG	98
EKG40_03390	deaminase	CGCTGGGTATTTCGTTGAT	GAACCCAGGCAGACATTC	97
EKG40_15900	deaminase	TGATCGCGACTTCATGC	ATGATTTCGCCGTCCTG	102
EKG40_08555	benzoate 1,2-dioxygenase	TGAGTGGCTGGAGATGTATG	CGTACCAGATCAGCGAGATT	110
EKG40_02865	homogentisate 1,2-dioxygenase	ACGAATTCAGCAGTGAGG	CGGAGAACAGTTCGGTATAG	96
EKG40_27950	cytochrome P-450	GTCTTCCAGTCTCCCTACTAC	TTCTCGCGCATCAATCAC	100
EKG40_03995	catechol 1,2-dioxygenase	GGACAGAAACGAGTCCAAAC	GTCGCCAAACGATACTCTTC	101
EKG40_08570	catechol 1,2-dioxygenase	CTGGAAAGCCGTCAACTAC	TCAGCAGGTCGAGGTAAT	98
EKG40_25940	catechol 1,2-dioxygenase	CAGAGGCCAGCACATATTC	AATCGTCGTGCAGGTATTG	100
EKG40_13720	gyrase	GTGTACTGGCTCAAGACTTAC	AGCATGGTGGTGATGTATTC	110
